# The Genetic Landscape of Human Glioblastoma and Matched Primary Cancer Stem Cells Reveals Intratumour Similarity and Intertumour Heterogeneity

**DOI:** 10.1155/2019/2617030

**Published:** 2019-03-07

**Authors:** Chiara Pesenti, Stefania Elena Navone, Laura Guarnaccia, Andrea Terrasi, Jole Costanza, Rosamaria Silipigni, Silvana Guarneri, Nicola Fusco, Laura Fontana, Marco Locatelli, Paolo Rampini, Rolando Campanella, Silvia Tabano, Monica Miozzo, Giovanni Marfia

**Affiliations:** ^1^Division of Pathology, Fondazione IRCCS Ca' Granda Ospedale Maggiore Policlinico, Milano, Italy; ^2^Department of Pathophysiology and Transplantation, University of Milan, Italy; ^3^Laboratory of Experimental Neurosurgery and Cell Therapy, Neurosurgery Unit, Fondazione IRCCS Ca' Granda Ospedale Maggiore Policlinico, Italy; ^4^UOS Research Laboratories, Fondazione IRCCS Ca' Granda Ospedale Maggiore Policlinico, Milano, Italy; ^5^Laboratory of Medical Genetics, Fondazione IRCCS Ca' Granda Ospedale Maggiore Policlinico, Milan, Italy

## Abstract

Glioblastoma (GBM) is the most malignant human brain tumour, characterized by rapid progression, invasion, intense angiogenesis, high genomic instability, and resistance to therapies. Despite countless experimental researches for new therapeutic strategies and promising clinical trials, the prognosis remains extremely poor, with a mean survival of less than 14 months. GBM aggressive behaviour is due to a subpopulation of tumourigenic stem-like cells, GBM stem cells (GSCs), which hierarchically drive onset, proliferation, and tumour recurrence. The morbidity and mortality of this disease strongly encourage exploring genetic characteristics of GSCs. Here, using array-CGH platform, we investigated genetic and genomic aberration profiles of GBM parent tumour (*n* = 10) and their primarily derived GSCs. Statistical analysis was performed by using R software and complex heatmap and corrplot packages. Pearson correlation and *K*-means algorithm were exploited to compare genetic alterations and to group similar genetic profiles in matched pairs of GBM and derived GSCs. We identified, in both GBM and matched GSCs, recurrent copy number alterations, as chromosome 7 polysomy, chromosome 10 monosomy, and chromosome 9p21deletions, which are typical features of primary GBM, essential for gliomagenesis. These observations suggest a condition of strong genomic instability both in GBM as GSCs. Our findings showed the robust similarity between GBM mass and GSCs (Pearson corr.≥0.65) but also highlighted a marked variability among different patients. Indeed, the heatmap reporting Gain/Loss State for 21022 coding/noncoding genes demonstrated high interpatient divergence. Furthermore, *K*-means algorithm identified an impairment of pathways related to the development and progression of cancer, such as angiogenesis, as well as pathways related to the immune system regulation, such as T cell activation. Our data confirmed the preservation of the genomic landscape from tumour tissue to GSCs, supporting the relevance of this cellular model to test *in vitro* new target therapies for GBM.

## 1. Introduction

Glioblastoma (GBM) is the most common malignant primary tumour of the central nervous system (CNS). The standard first-line management for this cancer consists in maximum surgical resection, but its ability to deeply infiltrate makes complete resection quite impossible. GBM is characterized by rapid progression and invasion, cell infiltration, intense angiogenesis, resistance to radio- and chemotherapies, and high frequency of relapse. This aggressive behaviour is responsible for the poor prognosis, with a median survival of about 14 months and a 5-year survival rate of 5.1% [1]. GBM resistance to therapies and the high frequency of recurrence are mainly due to a subpopulation of tumourigenic stem-like cells, known as GBM stem cells (GSCs), able to hierarchically initiate, maintain, and spread the neoplasm. GSCs are self-renewing, pluripotent, highly proliferative, and genetically unstable. Unlike normal stem cells, which acquire a quiescence after DNA damage, GSCs express a plethora of proteins that promote cell survival [2].

Despite the advances in the GBM biology knowledge, comprising behaviour, molecular features, and the heterogeneous genetic landscape, only few targeted therapies have been developed and applied in clinics [3–5]. However, an increasing number of studies that exploit genomic approaches have identified genetic markers typical of glioma and useful for diagnosis. These evidence led the World Health Organization to introduce new diagnostic guidelines based upon molecular diagnosis in 2016 (WHO) [6]. Therefore, nowadays, GBMs are distinguished in IDH-wildtype or IDH-mutated, depending on the presence of recurrent hotspot mutations in isocitrate dehydrogenases IDH1 and IDH2. Notably, large sample studies reported that IDH mutation is found in the majority of secondary GBMs (70-80%) and only rarely in primary GBMs [7]. GBMs are well characterized also from an epigenetic point of view and the DNA-hypermethylated phenotype, called CpG island methylator phenotype (CIMP), has been correlated to a good prognosis [8]. In particular, the most established improvement in predicting GBM patients' outcome is the methylation of the O6-Methylguanine-DNA methyltransferase (*MGMT*) promoter, which has been associated to a positive response to temozolomide [9, 10]. However, the constant improvement in genetic characterization of GBMs is still failing to be translated to clinical practice, suggesting that other discovery paradigms should be considered. At DNA level, GBMs are usually characterized by high levels of genomic instability. The GBM genome shows many copy number alterations (CNAs) that have been catalogued by computational methodologies [11–13]. These studies collectively have identified frequently amplified genes such as EGFR, MET, PDGFRA, MDM2, PIK3CA, CDK4, and CDK6 and deleted genes including CDKN2A/B, PTEN, and RB1. During the last years, array comparative genomic hybridization (array-CGH) has successfully contributed to improve the detection rate of genomic unbalances and alterations in cancer and to correlate recurrent CNAs to cancer pathomechanisms and prognosis [14, 15]. Considering the importance of CNA data, we focused on the comparison between genetic and genomic aberration profiles of GBM tumour masses and their primarily derived GSCs, investigating if GSC population harbours typical alterations different from the tumour bulk. Since GSCs are widely exploited in *in vitro* disease studies, uncovering dissimilarities with the corresponding tumour masses could be useful to set more reliable GBM models and to test new potential targeted therapies that could be effective also on the cell populations that are therapy resistant. In order to address these questions, we analysed recurrent hotspot mutations and performed array-CGH to compare CNAs between GBM and their derived GSCs at whole genome level.

## 2. Materials and Methods

### 2.1. Study Population

Ten GSCs were derived from matched primary (*n* = 9) and recurrent (*n* = 1) GBMs provided by the Neurosurgery Unit of the Fondazione IRCCs Ca' Granda Ospedale Maggiore Policlinico (Milan), according to the ethical requirements of the institutional committee on human experimentation.

Patients recruited between January 2014 and May 2016 were randomly included in the study, after informed consent was signed. The histological analyses were based on the 2016 WHO criteria for CNS tumours [3]. Demographic and clinical data are reported in Table 1.

### 2.2. GBM Sample Collection

GBM sample processing was performed as previously reported [16]. Briefly, after each surgical intervention, the tumour specimen was collected and maintained in Dulbecco's modified Eagle's medium: Nutrient Mixture F-12 (DMEM/F12, Thermo Fisher, Waltham, Massachusetts) containing 1% penicillin/streptomycin (Thermo Fisher) at 4°C, for up to 24 h. An aliquot of tissue was fixed in a 4% paraformaldehyde (PFA) solution (Sigma-Aldrich, Basel, Switzerland) in Dulbecco's phosphate-buffered saline (D-PBS) and subsequently embedded in paraffin (Sigma-Aldrich). Another aliquot was firstly mechanically minced with a surgical scalpel and then enzymatically digested with 0.625Wu/mL Liberase Blendzyme 2 (Roche, Mannheim, Germany) for 1 h at 37°C [17]. A cell suspension was obtained after passing dissociated through a 0.70 *μ*m pore size filter (Thermo Fisher).

### 2.3. Primary GSC Isolation

GSCs were isolated as previously described [17]. Briefly, GBM cells were cultured in the appropriate medium for isolation of GSCs, consisting of DMEM/F12 supplemented with 20 ng/mL epidermal growth factor (EGF) (R&D Systems, Minneapolis, USA), 10 ng/mL fibroblast growth factor-2 (bFGF) (PeproTech Inc., Rocky Hill, NY, USA), and 1% penicillin/streptomycin. Cells were cultured in a humidified incubator with 5% CO_2_, 5% O_2_ at 37°C, and split once a week. At each passage (P), GSC neurospheres were dissociated with Tryple Select (Gibco, Grand Island, NY, USA) and Trypan Blue dye exclusion assay (Gibco) was used to assess cell viability. Cells were routinely observed with an inverted phase-contrast microscope (Nikon Eclipse TE300; Nikon, Shinjuku, Tokyo, Japan), and images were acquired with a digital camera (Zeiss Axiovision, Zeiss, Oberkochen, Germany).

### 2.4. Immunophenotypic Analyses

Flow cytometric analyses were performed to assess the immunophenotypic profile of GSCs. The assay was conducted as previously described [17]. Briefly, each GSC line (5 × 10^4^ cell/tube) was incubated with phycoerythrin- (PE-) or fluorescein isothiocyanate- (FITC-) conjugated antibody to rate the expression of the following stemness/progenitor cell markers: CD15, CD31, CD45, CD133 (Miltenyi Biotec, Bisley, Surrey, UK), and CD90 (Millipore Temecula, CA, USA). Notably, 7-aminoactinomycin D (7-AAD, BD) was added to each tube to exclude dead cells from the analysis. After an incubation of 30 min at room temperature, washing and fixation with 4% PFA, immunomarked GSCs were scanned on a fluorescence-activated cell sorting (FACS) flow cytometer and analysed by CellQuest software (BD Biosciences, San Jose, CA). FlowJo software (Tree Star Inc., Ashland) was used to perform postprocessing analyses.

### 2.5. DNA Isolation

DNA from the FFPE tumour samples was obtained using a Biostic FFPE tissue DNA isolation kit (MO BIO Laboratories, Carlsbad, CA, USA), following manufacturer's instructions. DNA extraction was performed from FFPE tissue sections with at least 70% of tumour content, assessed by hematoxylin/eosin staining. DNA was extracted from GSCs, at a passage ranging from P4 and P10, using the QIAamp DNA Micro Kit (Qiagen, Hilden, Germany), according to manufacturer's protocol.

### 2.6. *IDH1*, *IDH2*, and *TERT* Mutation Analysis

The assessment of hotspot mutations in *IDH1*, *IDH2*, and *TERT* promoter was performed as previously described [18]. In brief, DNA was amplified by multiplexed primer mix targeting codon 132 and 172 of *IDH1* and *IDH2*, respectively, and position c.-124 and c.-146 of *TERT* promoter. PCR, SAP, and IPLEX reactions were conducted as described in manufacturer's protocol (Agena Bioscience, San Diego, CA, USA).

Samples were transferred to a SpectroCHIP (Agena Bioscience, San Diego, CA, USA) and analysed by mass spectrometry. The spectral profiles generated by MALDI-TOF mass spectrometry were evaluated using Typer v.4.0 software (Agena Bioscience, San Diego, CA, USA).

### 2.7. *MGMT* Promoter Methylation Evaluation

*MGMT* methylation was assessed both in FFPE tumour tissue and GSCs. DNA was modified with sodium bisulfite using the EZ DNA Methylation-Gold Kit (Zymo Research Corp., Irvine, CA). The methylation analysis was implemented as previously described [10, 19]. PCR was performed on at least 20 ng of bisulfite-treated DNA and about 10 pmol primers. Quantitative DNA methylation analysis was carried out on the Pyro Mark ID instrument using Pyro Gold Reagents (Qiagen) and 1 pmol of sequencing primer. Methylation data were analysed by the Q-CpG software v1.9 (Qiagen) and the levels of methylation of each sample are represented by the mean of the methylation percentages at each CpG site of the investigated region.

### 2.8. Array-CGH

Array-CGH analysis was performed as previously described [19], using 60-mer oligonucleotide probe technology (SurePrint G3 Human CGH 8 × 60 K, Agilent Technologies, Santa Clara, CA, USA), according to manufacturer's instructions. Agilent Feature Extraction was exploited to generate raw data, which were further analysed using Cytogenomics 2.7 with the ADAM-2 algorithm (Agilent Technologies, Santa Clara, CA, USA). In order to increase the accuracy of the results, the Diploid Peak Centralization algorithm was applied. A minimum of three consecutive probes/region was considered as filter. The threshold for genomic deletion is *x* = −1; the threshold for genomic gain is *x* = +0.58. Notably, in a mosaic scenario, the threshold is between -1 and 0 for deletions and between 0 and +0.58 for duplications [20]. Amplifications and homozygous deletions are considered with threshold >+2 and <-1, respectively. Variants reported as population variants in the public databases were not listed. Genomic coordinates are according to the build 37 assembly (March 2009) of the Human Genome Reference consortium (GRch37/hg19).

### 2.9. Statistical Analysis

Statistical analysis has been performed by using R software and complex heatmap [21] and corrplot packages (https://github.com/taiyun/corrplot) were exploited to figure out the data. Pearson correlation was used to compare genetic alteration profiles in matched pairs of GBM and derived GSCs. In order to group similar genetic profiles among samples, *K*-means algorithm was performed. Moreover, we used the chi-square test to compare *MGMT* methylation levels of GBM with the matched GSC samples. GO-Term enrichment analysis was developed using WebGestalt (http://www.webgestalt.org/option.php).

## 3. Results

### 3.1. Clinical Features of Patients Affected by GBM

Our GBM cohort has an even proportion of male and female patients with a mean age at diagnosis of 59 years (ranging from 36 to 82 years old). All tumours had been examined and classified according to the WHO guidelines and all GBMs were IDH-wildtype; GBM of patient (Pt) 3 is the first relapse. The observed overall survival (OS), in terms of time between the date of surgery and the date of death, varies between 2 and 54 months, with a mean value of 14.2 and a median value of 11.5 months. The Karnofsky Performance Score (KPS) was performed during hospitalization, before surgery, and assessed between 60% and 90%. As a cellular marker for proliferation, the Ki-67 protein was evaluated by immunohistochemistry, in a range between 15% and 70%.

### 3.2. GSCs Differ in Stemness Marker Expression

**Figure 1:**
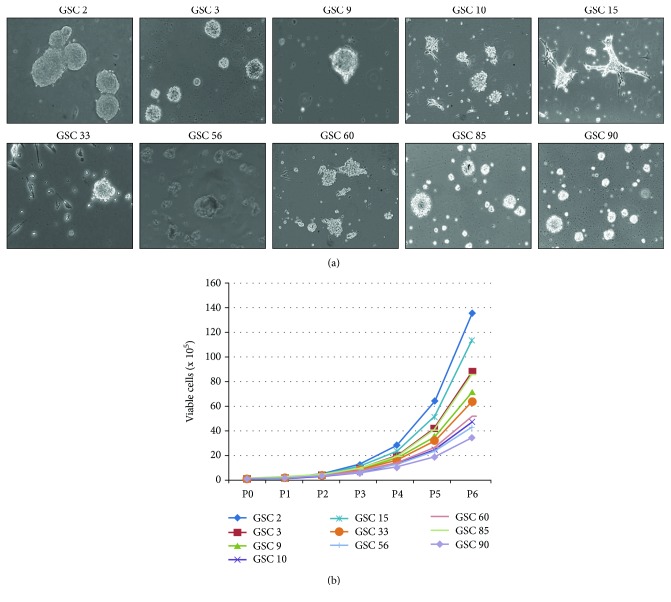
GSC isolation and propagation. (a) Representative images of GSCs of each patient, captured at passage 5. Magnification 10x with an inverted phase-contrast microscope. (b) Proliferation curves of GSCs. Each passage was done every 5 days.

About 30% of GSC lines experienced both an adherent and a floating phase (GSC 15, GSC 33, and GSC 56), still maintaining stemness properties (Figure 1(a)). Notably, estimating the number of viable cells from P0 to P6 (30 days), we observed different growth rates, thus each GSC line shows a specific proliferation curve, anyhow growing with an exponential rate (Figure 1(b)). Analysing GSCs, at a passage ranging from P4 to P10, by flow cytometry, we determined the expression of CD133, CD90, CD15, CD31, and CD45 as stem/progenitor cell markers, reporting different expression patterns among patients. As shown in Table 2, all the investigated stemness markers were expressed in our GSCs, with CD90 as the highest one. Notably, performing flow cytometry at higher passages, we observed that marker expression remains rather stable in 90% of GSCs, with a variability of about 5-10%.

### 3.3. Recurrent Hotspot Alterations

We investigated the most frequently reported genetic alterations for GBM IDH-wildtype in all GBM-GSC-matched couples at a passage ranging from P4 to P10, as in correspondence of the flow cytometric analysis. The presence of the hotspot mutations c.-124C>T and c.-146C>T in the promoter region of *TERT* was evaluated. As shown in Table 3, 8 out of 10 tumours were mutated in *TERT*, 6 harboured the c.-124C>T mutation (Pt 2, Pt 10, Pt 15, Pt 56, Pt 85, and Pt 90), while 2 had the c.-146C>T mutation (Pt 9 and Pt 60). For all samples, the same alteration found in the GBM was present also in the GSCs, confirming the driver function of these mutations. Moreover, the high prevalence of mutated cases in our small cohort of patients is concordant with the one reported by previous large studies on GBM IDH-wildtype [22, 23], further corroborating the relevance of such marker in this type of glioma.

We analysed the *MGMT* promoter methylation, which is the principal prognostic factor in primary GBM [24]. One out of 10 GBMs (Pt 90) was substantially hypermethylated at this promoter region, while Pt 10, Pt 15, and Pt 33 were only partially methylated; the remaining GBMs were nonmethylated (Table 3). Analogously to *TERT* mutations, the methylation levels of *MGMT* promoter were fairly concordant between GBM and GSCs of each patient. Particularly, nonmethylated GBMs showed nonmethylated GSCs. Regarding the methylated cases, Pt 10 and Pt 33 showed almost equal methylation percentages in the two components, considering the 5% of sensitivity limitation of the pyrosequencing technique [25], while Pt 15 and Pt 90 displayed more different values (*p* value = 1.12% for Pt 15 and 1.96% for Pt 90). In particular, Pt 90 showed an increment in methylation in GSCs (86% vs. 58% in the tumour, Table 3), which could be explained considering that GBM is more heterogeneous and nonneoplastic cells could partially alter the methylation level. Conversely, GSC 15 methylation is lower than GBM 15 (14% vs. 31% in the tumour, Table 3). In order to deepen the differences in methylation levels in Pt 15 and Pt 90, we verified the presence of deletions encompassing the *MGMT* locus (on chromosome 10q26.3) (Table 3) and both GBM 90 and GSC 90 exhibited a monosomy of chromosome 10 with a mosaic distribution. It means that not all cells harbour a deletion of one *MGMT* allele and that the heterogeneity is more evident in tumour bulk. However, Pt 15 displays deletions of *MGMT* with a mosaic pattern only in the GBM component, while the GSCs have no genomic aberration at this locus, which could partially explain the lower methylation levels in GSCs (Table 3).

### 3.4. Array-CGH Profiles

**Figure 2:**
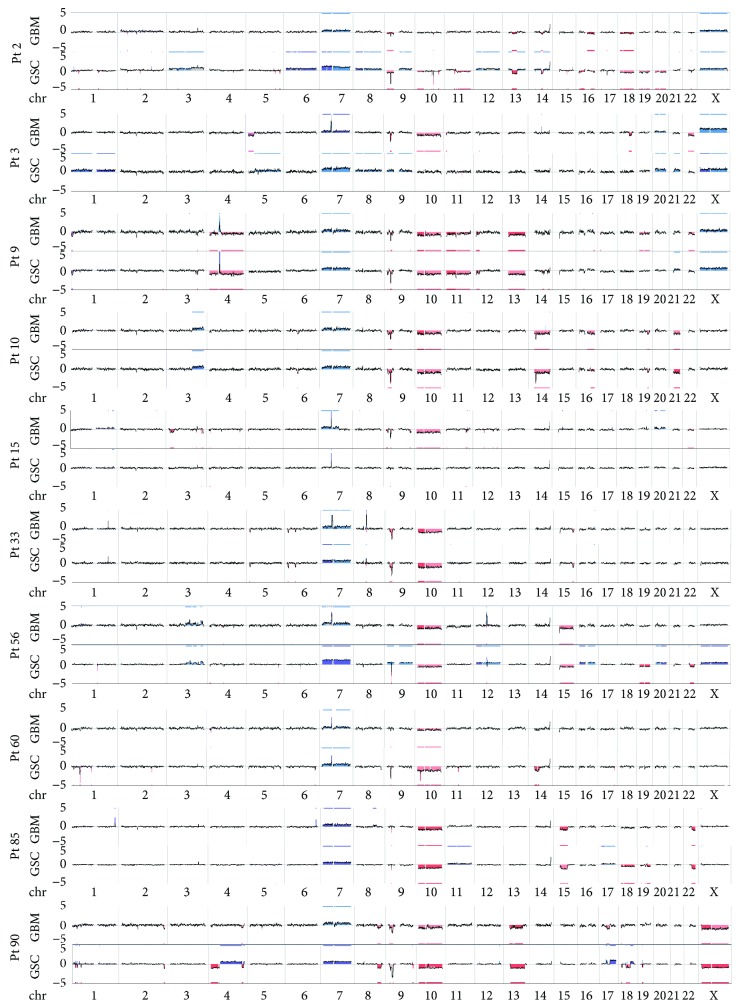
Genome view of GBM and GSCs of each patient displaying high intrapatient similarity and interpatient discordance. Gains or losses of chromosomal regions are computed by comparing signal intensity in GBM and GSCs with the background signal of the reference and then applying a segmenting and smoothing algorithm. Line plots show chromosomal changes seen in GBMs; the *y*-axis indicates the log2 value ranging from -5 to 5; blue indicates gain/amplifications, red losses/deletions.

Array-CGH experiments confirmed the robust similarity between GBM mass and GSCs of each case (Figure 2) but also highlighted a marked variability among different cases. Common alterations, present in almost all tumours, were polysomy of chromosome 7, monosomy of chromosome 10, and homozygous 9p21 deletion.

**Figure 3:**
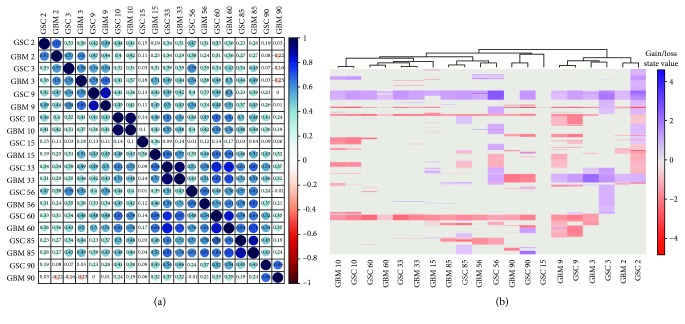
Hierarchical clustering results in our cohort of 10 patients highlighted the intrapatient similarity. (a) Correlation map reporting Pearson correlation values for each comparison. The bar on the left of the map indicates the color legend of the Pearson corr. values calculated for each couple of samples in the matrix. (b) Heatmap reporting Gain/Loss State for 21022 coding/noncoding genes (*y*-axis) in all samples (*x*-axis). Red and blue colors represent, respectively, losses and gains.

To measure the genomic relationship between GBM and derived GSCs, we performed Pearson correlation metric (Figure 3(a)). The mean Pearson correlation is 0.43, range -0.23/+1. By setting the correlation threshold to 0.65 (calculated from our data considering the mean correlation added to the standard deviation), we found that all patients, except for Pt 3, Pt 15, and Pt 56, showed a good correlation (Pearson correlation ≥ 0.65), denoting the concordance between GBM and matched GSC.

Notably, Pt 3 and Pt 56 had, respectively, Pearson corr. values of 0.58 and 0.64, thus below the threshold, but fairly close to it, whereas Pt 15 Pearson correlation was 0.36, corroborating the mentioned increase in dissimilarity between GBM and GSCs for this patient, which is already appreciable from the genome view reported in Figures 2 and 3(a).

Moreover, we obtained the *p* values of our correlation metric using cor.mtest function in R that performs the significance test for each pair of correlations; *p* values < 0.05 indicate a significant correlation between samples. Notably, all *p* values resulted <0.05, showing a significant correlation between GBM and GSC of each patient, also for Pt 15.

Noteworthy, Pt 15 has the lowest number of genomic aberrations among our cohort. This could be related to his long-term survival (OS 54 months), significantly higher than the mean GBM OS (14.2 months) and to his young age (36 yrs, with respect to the GBM patient median age of 59 yrs). Unfortunately, the fact that Pt 15 is the only patient with these characteristics, due to the great rarity of such high OS cases, prevented further considerations about his genomic pattern.

To further assess the intrapatient and interpatient correlation, we investigated the distribution of CNAs (gains and losses) in GBM and GSCs along genes (coding and noncoding). As reported in Figure 3(b), except Pt 15, all GBM-GSC-matched pairs are clustered together, highlighting the strong correspondence of GBM and GSCs in each patient. This analysis also quite demonstrated the divergence between GBM and GSCs of Pt 15, further corroborating the abovementioned considerations.

Taken together, the data show an increment of alterations in GSCs than in GBM, i.e., GSC 90 at chromosomes 4, 17, and 18 compared to the matched GBM 90 (Figure 1).

Therefore, to deepen the comparison of GBM and GSCs, we decided to focus on copy number patterns of well-characterized genetic markers in primary GBMs that could be also exploited for the development of new treatments: *EGFR*, *PDGFRA*, *MDM4*, and *MET* amplification and *CDKN2A*, *PTEN*, and *NFKB1* deletions (Table 3). We highlighted that our cohort of patients almost completely recapitulates the information reported in literature, ensuring that not selection biases were made.

All of our samples, except again Pt 15, displayed a complete polysomy of chromosome 7, encompassing both *EGFR* and *MET* genes. The chromosome 7 polysomy is considered a fundamental event driving GBM tumourigenesis. Indeed, it was present in almost all GBMs and derived GSCs together with the monosomy of chromosome 10, encompassing the *PTEN* locus and the focal deletion of chromosome 9p21 surrounding *CDKN2A* [26–29]. Regarding *EGFR*, it was further substantially amplified in 5 out of 10 samples (Pt 3, Pt 15, Pt 33, Pt 56, and Pt 60), comprehending also Pt 15 that had only the focal amplification of *EGFR* locus in both GBM and GSCs. It is noteworthy that 3 out of 5 *EGFR*-amplified GBM lost this marker in the derived GSCs (Pt 3, Pt 33, and Pt 56). Moreover, none of the samples had *MET* amplification, further supporting the rarity of this condition [4]. As for the polysomy of chromosome 7, almost all GBMs and GSCs harboured monosomy of chromosome 10, thus at least one allele of *PTEN* is deleted in all GBMs and matched GSCs, confirming the driver function of this alteration in gliomagenesis [26–29].

Another marker present in almost all samples was the deletion of chromosome 9p21 involving *CDKN2A*. It was present at homozygous status in the majority of patients, with some discordance between GBM and GSC, probably due to the heterogeneity of the samples, such as for GBM 56. Remarkably, Pt 3, the relapse sample, exhibited two opposite patterns of alteration at this locus in his GBM and GSCs, del and gain, respectively, (Table 3).

Finally, the other markers were present each in only one sample, confirming the low frequency of these alterations, particularly, Pt 9 (GBM and GSCs) lost *NFKB1* in a mosaic pattern and displayed the amplification of *PDGFRA*. Pt 33 (GBM and GSCs) instead was the only sample with *MDM4* amplification.

### 3.5. Clustering of Similar Genetic CNA Profiles in GBM and GSC Samples

**Figure 4:**
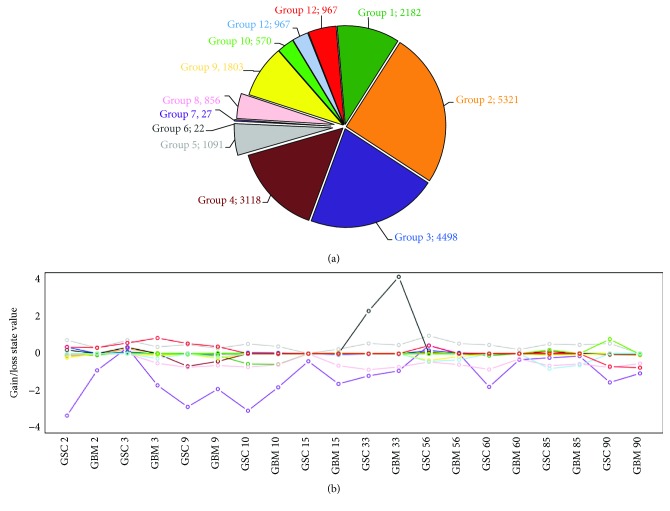
*K*-means results to cluster similar genetic CNA profiles in our GBM and GSC samples in 12 groups. (a) Pie chart reporting the number of genes gathered in each cluster according to *K*-means algorithm. (b) Centroid profiles for each cluster. Each line depicts the intracluster CNA mean values among samples (*x*-axis). Grey line represents the cluster 5 profile, containing genes amplified in all patients except GBM 90. Purple and pink lines are referred to clusters 7 and 8, containing genes deleted in all samples except for GSC 3 and GSC 56.

In order to further evaluate the genetic patterns, we used *K*-means algorithm to find genes with similar profiles among samples. We chose *k* = 12 because it maximizes the variance interclusters and minimizes the variance intracluster (Supplementary Material (available here)). Results are shown in Figure 4, reporting the average genetic alteration profile line for each cluster. All clusters seemed to profile randomly among samples, except clusters 5, 7, and 8. Cluster 5 (grey area and grey line in Figures 4(a) and 4(b), respectively) contains 1091 genes (including *EGFR*), which were amplified on average in all samples, except GBM 90. Clusters 7 (purple area and line in Figures 4(a) and 4(b)) and 8 (pink area and line in Figures 4(a) and 4(b)) contain, respectively, 27 and 856 genes (encompassing *CDKN2A* and *PTEN*, respectively), which were deleted in about 90% of samples.

We performed GO-Term enrichment analysis using WebGestalt tool to investigate the pathways of genes involved in these aberrations and found that there is an impairment of pathways related to cancer development and progression (Supplementary Material). In particular, we identified clear distinctive pathways included in the three clusters analysed. In cluster 5, we identified genes involved in the new vessel formation and in nitrogen and oxygen response. In cluster 7, the main identified pathways are related to the immune response as “regulation of cytokine-mediated signaling pathway,” whereas in cluster 8, we identified genes associated at two distinctive pathways, metabolic process pathway and interestingly brain development.

## 4. Discussion

GBM is highly heterogeneous and resistant to conventional chemo- and radiotherapies and several recent studies suggested that it is driven and maintained by GSCs. The development of more personalized therapies is still urgent and *in vitro* models able to reliably recapitulate the original tumour are yet under discussion. Here, we focused on the characterization of the genomic patterns of GBM masses and respective isolated GSCs in 10 primary GBM patients to additionally establish the reliability of GSCs as *in vitro* model and to search for genetic features typical of GSCs and able to explain the resistance and aggressiveness of these cells.

Remarkably, the characterization of primary GSCs in the analysed cases revealed patient-specific profiles, both in terms of growth rate and surface stemness marker expression. Particularly, we observed a high interpatient variability and a peculiar immunophenotypic profile in each GSC population, in accordance with the well-known heterogeneity of GBMs [30]. Interestingly, our cytofluorimetric analysis revealed that all GSCs were all positive, but at different levels, for the markers of stemness and endothelial progenitors: CD15, CD133, CD90, CD31, and CD45.

Although some authors have demonstrated that *in vitro* growth of GSCs and their stemness marker expression represents a significant predictor of clinical outcome [31–33], the real association between the biological features of GSCs and GBM profile has yet to be established.

From a genomic perspective, the typical genetic features of primary GBMs were markedly concordant between GBM/GSC couples, confirming the high similarity between the tumour mass and its respective GSC population. Regarding *TERT* mutations, 8 out of 10 GBMs and matched GSC cases were mutated in the promoter region. Of note, *TERT* mutations have a prognostic role in gliomas, being a negative prognostic factor in primary GBM without *MGMT* methylation [34]. In our cohort, two cases were methylated at the *MGMT* promoter (Pt 15 and Pt 90), but unfortunately, the number of samples included prevented the possibility to perform statistically significant analyses about the link between *TERT* promoter mutations, *MGMT* methylation, and patients' survival. Nevertheless, this was beyond the purposes of this study. The almost similar levels of *MGMT* promoter methylation between GBM and GSCs suggested that also this epigenetic marker is conserved in the GSC population, which is usually responsible for the high frequency of recurrence in this type of tumour. This evidence is in accordance with the fact that generally the level of methylation between primary GBMs and respective relapses is fairly comparable [35]. Moreover, the increased methylation levels of GSC 90 could be explained by the fact that it is a more homogeneous sample than the whole GBM mass [3]; in addition, a sample heterogeneity cannot be excluded, as already suggested [3, 36]. Conversely, the decreased methylation level in GSC 15 compared to GBM 15 could be due to the absence of deletions at this locus. Nevertheless, the methylation levels in both GSC 15 and GSC 90, along with the presence of mosaic deletions of *MGMT* in GSC 90, suggest that also the GSC population is heterogeneous and not all cells are *MGMT* methylated.

Almost all GBM and GSC couples had polysomy of chromosome 7, monosomy of chromosome 10 (*PTEN*), and deletions at chromosome 9p21 (*CDKN2A*) that are typical features of primary GBM, essential for gliomagenesis and usually conserved in the whole GBM mass [13, 26, 28, 29, 37]. The only two patients showing different patterns of these markers between GBM and GSCs were Pt 3 and Pt 15. Pt 3 showed opposite molecular features at the 9p21 region between GBM (9p21 loss) and GSCs (9p21 gain). This GBM is the first tumour recurrence, meaning that the patient had been already treated and this could have affected the genomic configuration of the GBM and/or GSCs, although the Pearson corr. is quite close to the threshold of 0.65. Another possible mechanism to explain the peculiar molecular profile of Pt 3 could be the “parallel evolution” model proposed by Baysan et al. [26]. By this theory, different clones could evolve separately from a common ancestor, giving rise independently to fairly equal genomic patterns between primary and recurrent tumours.

Pt 15 is the only young long-term survival patient included in the study and he has substantially less genomic aberration both in GBM and in GSCs. In details, Pt 15 showed the amplification of *EGFR* without the polysomy of the chromosome 7 in both GBM and GSCs, the monosomy of chromosome 10 only in GSCs, and the deletion of *CDKN2A* in a mosaic fashion in GSCs. The asset of these GBM peculiar markers in long-term GBM survivors is still discussed; therefore, it is quite complex to define their association with overall survival [1, 38–40].

Regarding *EGFR* amplification, 5 out of 10 analysed cases exhibit this alteration in GBM, but only 2 of them retained it in GSC population. This could be due to the negative selective pressure induced by EGF addition to the medium, as described by [41, 42]. However, this phenomenon is still controversial; indeed, not all GSCs derived from *EGFR*-amplified GBM lack the amplification of this marker, as reported in several studies [43–45].

The polysomy of chromosome 7 involves also the *MET* locus, which is never amplified in our cohort, neither in the cases without *EGFR* amplification. Indeed, *MET* is usually overexpressed in mesenchymal/proneural subgroup of GBMs *EGFR*-wildtype and *PTEN*-lost [46], thus configuring as an alternate way to deregulate the tyrosine kinase signaling pathway. Other alternative mechanisms to impair the signaling pathway mediated by tyrosine kinases are the *PDGFRA* amplification and the *NFKB1A* deletion [4, 47].

Pt 9 displayed a normal pattern of *EGFR* and harbours both *PDGFRA* amplification and *NFKB1A* deletion in GBM and GSCs. Finally, only GBM and GSCs of Pt 33 harboured *MDM4* amplification that is an alternative and quite infrequent route to inactivate p53 [4].

Therefore, almost all samples show concordance between GBM and GSCs, as demonstrated by the Pearson correlation analysis (Pearson corr.≥0.65), corroborating the fact that GSCs resemble their parental tumour at a genomic level [45, 48]. Besides this, the heatmap highlights the interpatient variability between GBM and GSC, further supporting the high heterogeneous behaviour of this type of tumour.

The slight intrapatient genomic differences between GBM and GSCs, and particularly the partial enrichment of alterations in GSCs, could be related to the heterogeneity of GBM and that some low frequent alterations could be masked by the presence of nonneoplastic cells in the analysed specimens. Nevertheless, the presence of these dissimilarities could also be due to the *in vitro* growing conditions of GSCs. Indeed, even if different GSCs did not acquire *in vitro* identical genomic alterations under the same selective pressure, it is anyway possible that *in vitro* culture could select small subpopulations of cells in the parental GBM mass harbouring alterations not detectable in the tumour bulk. Moreover, it was recently demonstrated that discordant inheritance of chromosomal and extrachromosomal DNA elements between primary tumour mass, neurosphere cultures, and xenografts exists and is strictly related to the dynamic GBM evolution [44]. Therefore, on the one side, elevated concordance between GSCs and the primary tumour masses corroborates the use of these as reliable *in vitro* and *in vivo* models of GBM; on the other side, the dissimilarities could be useful to understand clonal evolution of the tumour and to test new targeted treatments. Indeed, the *K*-means algorithm identified three clusters of genes equally altered among the GBM and GSC samples. These three clusters involved the already described genes *EGFR*, *CDKN2A*, and *PTEN*. However, besides these loci, GO-term enrichment analysis pointed out defects in other genes, with similar CNA profiles among GBM/GSC couples, encompassing pathways essential for the development and progression of cancer, such as pathway related to angiogenesis, as well as pathways related to the immune system regulation. This last point is particularly interesting, considering the promising advances in immunotherapies.

Despite the limitation of our work, related to the small number of patients analysed, our findings contribute to add a little piece to the complicated puzzle of GBM. The detailed analyses of genomic aberrations in GSCs further testify that they represent a good model since they maintain the main genetic markers of GBM. Indeed, as GSCs are patient-specific and radio- and chemoresistant, they represent a challenging target for therapies. In the era of precision medicine, indeed, a better understanding of the GSC molecular landscape is pivotal to design effective target therapies for GBM.

## Figures and Tables

**Table 1:** Clinicopathological characteristics of the patients enrolled in the study.

Patient ID	Sex	Age (yrs)	Dx	OS	KPS	Ki67
Pt 2	M	60	GBM	4	60%	15%
Pt 3	F	50	GBM(R)	16	70%	40%
Pt 9	F	82	GBM	5	60%	15%
Pt 10	F	63	GBM	5	90%	35%
Pt 15	M	36	GBM	54	80%	60%
Pt 33	F	65	GBM	11	90%	70%
Pt 56	M	75	GBM	2	60%	40%
Pt 60	M	49	GBM	16	80%	70%
Pt 85	M	59	GBM	12	80%	30%
Pt 90	F	58	GBM	17	90%	30%

Pt: patient; M: male; F: female; yrs: years; Dx: diagnosis; R: relapse; OS: overall survival expressed in months; KPS: Karnofsky Performance Score.

**Table 2:** Stemness marker analyses in GSCs revealed different expression patterns among GBM patients.

Sample	Passage	CD15	CD31	CD34	CD45	CD133	CD90
GSC 2	10	+	+++	+	+	+++	+++
GSC 3	4	+	+++	+	+	+++	+
GSC 9	4	+	+++	++	+	+	+
GSC 10	4	+	+	+	++	+	+++
GSC 15	4	+	++	++	+	++	+++
GSC 33	4	++	+	++	++	+++	+++
GSC 56	4	+	+	+	+	++	+++
GSC 60	5	+	+	++	+	+	+++
GSC 85	4	+	++	++	+	++	++
GSC 90	6	+	+	+	+++	+++	+++

+: 0-33%; ++: 34-66%; +++: 67-100%.

**Table 3:** Genetic profile of the main markers of IDH-wildtype GBM in our cohort of GBMs and GSCs.

Gene/ID		Pt 2	Pt 3	Pt 9	Pt 10	Pt 15	Pt 33	Pt 56	Pt 60	Pt 85	Pt 90	% tumours altered^∗^
*TERT* promoter mutation	GBM	c.-124C>T	WT	c.-146C>T	c.-124C>T	c.-124C>T	WT	c.-124C>T	c.-146C>T	c.-124C>T	c.-124C>T	72-90%
	GSC	c.-124C>T	WT	c.-146C>T	c.-124C>T	c.-124C>T	WT	c.-124C>T	c.-146C>T	c.-124C>T	c.-124C>T	

*MGMT* methylation	GBM	5%	4%	4%	12%	31%	15%	3%	2%	2%	58%	40-50%
	GSC	4%	2%	3%	9%	14%	20%	3%	2%	1%	86%	

*MGMT* CN	GBM	NA	LOSS	LOSS	LOSS	LOSS	LOSS	LOSS	LOSS	LOSS	LOSS	/
	GSC	NA	NA	LOSS	LOSS	NA	LOSS	LOSS	LOSS	LOSS	LOSS	

*EGFR* CN	GBM	GAIN (4)	AMP	GAIN	GAIN (4)	AMP	AMP	AMP	AMP	GAIN	GAIN	35-45% amplified
	GSC	GAIN	GAIN	GAIN	GAIN	AMP	GAIN	GAIN	AMP	GAIN	GAIN	

*PTEN*/10q CN	GBM	LOSS	LOSS	LOSS	LOSS	LOSS	LOSS	LOSS	LOSS	LOSS	LOSS	75-95% deleted
	GSC	LOSS	NA	LOSS	LOSS	NA	LOSS	LOSS	LOSS	LOSS	LOSS	

*CDKN2A* CN	GBM	LOSS	DEL	DEL	DEL	DEL	DEL	NA	LOSS	DEL	DEL	35-50% deleted
	GSC	DEL	GAIN	DEL	DEL	LOSS	DEL	DEL	DEL	DEL	DEL	

*NFKB1* CN	GBM	NA	NA	LOSS	NA	NA	NA	NA	NA	NA	NA	25% deleted
	GSC	NA	NA	LOSS	NA	NA	NA	NA	NA	NA	GAIN	

*PDGFRA* CN	GBM	NA	NA	AMP	NA	NA	NA	NA	NA	NA	NA	13% amplified
	GSC	NA	NA	AMP	NA	NA	NA	NA	NA	NA	GAIN	

*MDM4* CN	GBM	NA	NA	NA	NA	NA	AMP	NA	NA	NA	NA	7% amplified
	GSC	NA	GAIN	NA	NA	NA	AMP	NA	NA	NA	NA	

*MET* CN	GBM	GAIN	GAIN	GAIN	GAIN	NA	GAIN	GAIN	GAIN	GAIN	GAIN	4% amplified
	GSC	GAIN	GAIN	GAIN	GAIN	NA	GAIN	GAIN	GAIN	GAIN	GAIN	

^∗^Percentage of mutated tumours as defined by the reported references. CN: copy number; NA: not altered; DEL: log2 values <-1; LOSS: log2 values <0 and >-1; GAIN: log2 values >0 and <2; AMP: log2 values >2.

## Data Availability

The data used to support the findings of this study are available from the corresponding author upon request.

## Supplementary Materials

Supplementary Materials Supplementary Material: an Excel File in which there are reported lists of genes included in *n* = 12 clusters, grouped by *K*-means algorithm, with the relative location, values, and GO-Term enrichment analysis performed by WebGestalt. Moreover, all the output files achieved by WebGestalt are included.
